# Identification and thermochemical analysis of high-lignin feedstocks for biofuel and biochemical production

**DOI:** 10.1186/1754-6834-4-43

**Published:** 2011-10-21

**Authors:** Venugopal Mendu, Anne E Harman-Ware, Mark Crocker, Jungho Jae, Jozsef Stork, Samuel Morton, Andrew Placido, George Huber, Seth DeBolt

**Affiliations:** 1 Department of Horticulture, University of Kentucky, 1100 Nicholasville Road, Lexington, KY 40546, USA; 2 Center for Applied Energy Research, University of Kentucky, 2540 Research Park Drive, Lexington, KY 40511, USA; 3 Department of Chemical Engineering, University of Massachusetts, 686 North Pleasant Street, Amherst, MA 01003, USA

**Keywords:** biofuels, catalytic fast pyrolysis, bio-oil, lignocellulose, endocarp, bioenergy

## Abstract

**Background:**

Lignin is a highly abundant biopolymer synthesized by plants as a complex component of plant secondary cell walls. Efforts to utilize lignin-based bioproducts are needed.

**Results:**

Herein we identify and characterize the composition and pyrolytic deconstruction characteristics of high-lignin feedstocks. Feedstocks displaying the highest levels of lignin were identified as drupe endocarp biomass arising as agricultural waste from horticultural crops. By performing pyrolysis coupled to gas chromatography-mass spectrometry, we characterized lignin-derived deconstruction products from endocarp biomass and compared these with switchgrass. By comparing individual pyrolytic products, we document higher amounts of acetic acid, 1-hydroxy-2-propanone, acetone and furfural in switchgrass compared to endocarp tissue, which is consistent with high holocellulose relative to lignin. By contrast, greater yields of lignin-based pyrolytic products such as phenol, 2-methoxyphenol, 2-methylphenol, 2-methoxy-4-methylphenol and 4-ethyl-2-methoxyphenol arising from drupe endocarp tissue are documented.

**Conclusions:**

Differences in product yield, thermal decomposition rates and molecular species distribution among the feedstocks illustrate the potential of high-lignin endocarp feedstocks to generate valuable chemicals by thermochemical deconstruction.

## Background

Plant cell walls are extracellular composites that constrain the internal turgor pressure of plant cells, facilitate directional growth of cells and determine plant form and function. The cell wall is composed primarily of cellulose, lignin, hemicellulose and pectins as structural biopolymers and an abundance of highly glycosylated proteins. As individual components, the ratio of different cell-wall components varies from tissue to tissue and with the developmental stage of the plant [1]. The two most abundantly renewed carbon constituents of the biosphere, cellulose and lignin, represent attractive options for renewable fuels and products. Whereas cellulose has received much attention for its biochemical deconstruction capacity via enzymatic hydrolysis [1,2] or thermochemical deconstruction [3,4], lignin is often viewed as a waste product because of problems in its structural diversity and heterogeneity, which pose challenges to deconstruction [5-9]. Despite these challenges, lignin contains structural units that could serve as a source of fuels and high-value chemicals if means can be found to free these structural units from the polymer. Lignin is formed by a set of three precursor alcohols from the phenylpropanoid pathway (*p*-coumaryl alcohol, coniferyl alcohol and sinapyl alcohol) through a series of oxidation steps [10-12]. Lignification changes the biophysical properties of the plant cell and tissue type and has often been described as increasing structural integrity and providing waterproofing. Several fruits classified as drupes have heavily lignified endocarp that acts protects against physical damage and provides general biotic and abiotic stress tolerance [13].

Recently, the focus within the biofuels community has begun to shift from alcohol production to the production of hydrocarbon biofuels by thermochemical deconstruction. Such fuels are replacements for gasoline, diesel and jet fuel, and, given that they can function as drop-in fuels, they are far more attractive than ethanol for existing internal combustion engines [14]. The molecular makeup of bio-oil depends on the inherent composition of the biomass and pyrolytic conditions. In principle, the oxygenates resulting from the oxidative deconstruction of lignin can be either deoxygenated to produce hydrocarbons (for example, by (1) hydrodeoxygenation or hydrolysis and (2) fast pyrolysis [4]) or first subjected to separation to recover high-value chemicals that are present, such as phenols and cresols. Similar considerations have been applied to the upgrading of bio-oils obtained from biomass by fast pyrolysis. Indeed, simple water addition to pyrolysis oil results in its fractionation into a water layer containing mainly light oxygenates (derived from carbohydrates and, to a limited degree, from lignin) and water-insoluble, mostly lignin-derived oligomeric (aromatic) compounds [4,15]. Although lignin (based on its low oxygen content) appears to be a promising feedstock for biofuel production, significantly more research is needed to develop efficient conversion technologies for lignin-derived feedstocks. Moreover, suitable feedstocks should be identified and characterized to facilitate the development of these processes. The objective of this study was to identify and examine feedstocks that possess naturally high lignin content by means of thermochemical deconstruction. Preliminary experiments were performed to examine the pyrolytic characteristics of the various feedstocks and to estimate the potential of these feedstocks to produce bioelectricity, biofuel and high-value chemicals.

## Results

### Identification of plant feedstocks possessing high lignin content

**Figure 1:**
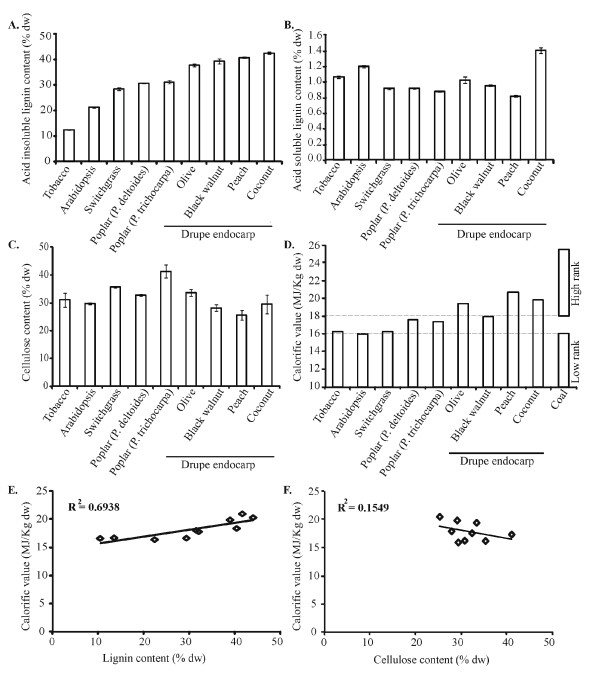
**Compositional characterization of biomass-based feedstocks**. The acid-insoluble lignin content of various feedstocks **(A)**, acid-soluble lignin **(B) **and acid-insoluble glucose **(C) **expressed as percentage content per unit dry weight (DW). Each feedstock was examined for calorific value **(D) **and compared with values for low-grade and high-grade coal. Pairwise comparison of lignin **(E) **or cellulose **(F) **with calorific values and corresponding *R*^2 ^values for the slope of the trend line. Feedstocks examined included high-lignin drupe endocarp tissue (from peach, olive, walnut and coconut), perennial grasses such as switchgrass (*Panicum virgatum*), woody biomass feedstocks such as Poplar (*Populus deltoides *and *Populus trichocarpa*) and leafy crops such as tobacco (*Nicotiana benthamiana*) and *Brassica *(*Arabidopsis thaliana*).

To identify high-lignin renewable plant-derived feedstocks, we posed two primary constraints on candidate feedstocks. First, we wanted to capture the breadth of lignification across a diversity of plant families, which resulted in the inclusion of *Arabidopsis *as an annual *Brassica *(Cruciferae), despite its not being a bioenergy crop candidate. Second, we sought to identify plants that already have existing value in their production, being food crops, plantation crops, horticultural crops or proposed bioenergy crops. We examined the aerial portion of switchgrass (*Panicum virgatum*) or Poplar stem (*Populus deltoides *and *P. trichocarpa*) and also included *Nicotiana benthamiana *stem and a range of fruit endocarp waste derived from horticultural crops grown abundantly worldwide, including olive (*Olea europaea*), black walnut (*Juglans nigra*), coconut (*Cocos nucifera*) and peach (*Prunus persica*) as characteristic species for the stonefruits [16-37] (Table 1). Our results establish that the upper-end potential of lignin in renewable plant feedstocks was found in endocarp tissue from horticultural crops. On average, these feedstocks comprised approximately 42% lignin, 30% cellulose and 1.5% ash (Table 1). In our own empirical determinations, we found that 44%, which occurred in the hardened endocarp of coconut (*C. nucifera*), was the highest lignin content (based on Klason lignin), although as reported in the literature, the content of peach and coconut endocarp can readily be upwards of 50% dry weight lignin [13,35]. By contrast, the low end of lignification was found in tobacco, which contained 13% lignin and 31% cellulose (Table 1 and Figures 1A and 1C). Two of the best-described biomass feedstocks with regard to their lignin content are switchgrass (*P. virgatum*), which ranges from 15% to 29% lignin, with an average of 22% [24], and the woody biomass of the short-rotation woody crop *Populus spp*. averages 25% lignin [28,38]. Biomass consists mainly of cellulose, lignin, hemicellulose, pectin and highly glycosylated proteins. Hence the remainder of the cell-wall material not characterized herein is proposed to be a composite of structural polymers and protein. Acid-soluble lignin did not show great variation among feedstocks (Figure 1B). In contrast to lignin content, the cellulose content of drupe endocarp tissue averaged 23% compared with the average switchgrass and woody crop cellulose content of 35%, and drupe endocarp tissue showed very low or undetectable amounts of ash (Tables 1 and 2).

**Table 1:** Biomass composition and calorific values of different feedstocks^a^

Common name	Scientific name	Ontology	Total lignin (%)	Cellulose (%)	Ash (%)	Calorific value (MJ/kg)	Reference
Tobacco^b^	*Nicotiana benthamiana*	Stem	13.6	31.1	7.0	16.3	
Tobacco^c^	*N. tabaccum*	Stem	17.4 to 21.0	26.0 to 30.9	15.0 to 30.0		[16-18]
*Arabidopsis*^b^	*Arabidopsis thaliana*	Stem	22.5	29.7	8.3	15.9	
*Arabidopsis*^c^	*Arabidopsis thaliana*	Stem	11.5 to 20.0	29.0 to 35.0	13.4		[19-21]
Switchgrass^b^	*Panicum virgatum*	Aerial	29.4	35.7	3.5	16.2	
Switchgrass^c^	*P. virgatum*	Aerial	15.0 to 29.8	33.5 to 46.1	4.6 to 5.7		[22-25]
Eastern cottonwood^b^	*Populus deltoides*	Stem	31.5	32.7	2.6	17.6	
Eastern cottonwood^c^	*P. deltoides*	Aerial	19.8 to 25.6	42.2 to 55.8	1.0		[22,26,27]
Black cottonwood^b^	*Populus trichocarpa*	Stem	32.1	41.4	2.7	17.3	
Black cottonwood^c^	*P. trichocarpa*	Aerial	25.2 to 28.9	40.3 to 45.0	1.7 to 2.0		[22,28,29]
Olive (DE)^b^	*Olea europaea*	Stone	39.0	33.7	1.2	19.4	
Olive (DE)^c^	*O. europaea*	Stone	20.6 to 26.5	29.8 to 34.4	0.01 to 0.7		[20,30]
Eastern black walnut (DE)^b^	*Juglans nigra*	Shell	40.4	28.2	1.4	17.9	
Black walnut (DE)^c^	*J. spp*.	Shell	18.6 to 28.5	54.0 to 60.2	0.6 to 1.1		[31]
Peach (DE)^b^	*Prunus persica*	Stone	41.6	25.6	2.9	20.5	
Peach (DE)^c^	*P. persica*	Stone	40.0 to 50.0	0.4 (FW)	0.7		[17,32-34]
Coconut (DE)^b^	*Cocus nucifera*	Shell	44.0	29.7	0.5	19.8	
Coconut (DE)^c^	*C. nucifera*	Shell	27.2 to 50.0	14.0 to 33.5	0.5 to 2.7		[35-37]

DE = drupe endocarp biomass; FW = biomass fresh weight; total lignin = acid-soluble lignin + acid-insoluble lignin. ^a^Biomass composition was analyzed to identify high-lignin feedstocks. Biomass analysis was based on the dry weight of the samples. Among the different feedstocks analyzed in this study, agricultural by-products (drupe endocarps) showed high lignin content. ^b^Data from this study. ^c^Data from the literature.

**Table 2:** Volatile, char and ash content of bioenergy feedstocks: high-lignin endocarp tissue versus perennial grasses and woody biomass feedstocks^a^

Feedstock	Volatile (wt%)	Char (wt%)	Ash (wt%)
High lignin (drupe endocarp)	77.0%	23.0%	None
Medium lignin 1 (switchgrass)	82.7%	17.3%	None
Medium lignin 2 (*Poplar*)	79.6%	20.4%	< 0.5%
Low lignin (tobacco)	78.9%	21.1%	3.9%
Moderate lignin (*Arabidopsis*)	76.7%	23.3%	3.1%

^a^The volatiles and char content were calculated from thermogravimetric analyzer plots. Samples were heated at a ramp rate of 150°C/minute to 800°C.

### Examination of bioelectricity or biofuel potential for high-lignin plant feedstocks

To establish the potential for bioelectricity generation among the feedstocks examined, the energy density of the feedstocks was calculated on the basis of the net calorific value of the dry biomass, which was determined using a bomb calorimeter. Endocarp biomass derived from peach (*P. persica*) showed the highest calorific value at 20.5 MJ/kg, followed by coconut (*C. nucifera*) endocarp (19.8 MJ/kg). In contrast, *Arabidopsis thaliana *stem biomass showed the lowest calorific value of 15.9 MJ/kg, documenting a variation of 4.6 MJ/kg between the lowest- and highest-value feedstocks (Table 1 and Figure 1D). The energy density of the drupe endocarp feedstocks overlapped with the range of values typical for high-rank coal (18.0 to 25.5 MJ/kg), indicating that the renewable bioelectricity potential relative to this form of fossil energy is comparable. The average calorific value of drupe endocarp biomass (olive, black walnut, peach and coconut) was 19.4 MJ/kg, representing a 20% higher "energy content" compared to switchgrass (16.2 MJ/kg). In comparison to woody bioenergy crops, drupe endocarp biomass had a 12% higher net energy value than the average of Poplar (*Populus trichocarpa *and *P. deltoides*) biomass (17.4 MJ/kg). On the basis of the average calorific value of the drupe endocarp (19.4 MJ/kg), endocarp biomass has the potential to produce 5.4 kWh/kg equivalent of bioelectricity.

To view how biomass composition influences net energy value, we generated pairwise interaction plots of calorific value (net energy value) and the endogenous lignin or cellulose content of the feedstock. These data revealed a positive interaction trend between lignification of the feedstock (percentage lignin content) and calorific value. Specifically, as lignin content increased, the net energy value increased at a rate of 1 MJ/kg for every 8.375% of lignin content. By contrast, no observable trend was determined for the interaction between energy content and cellulose content (Figures 1E and 1F).

### Thermogravimetry, differential thermogravimetry and gel permeation chromatography of high- and low-lignin feedstocks

**Figure 2:**
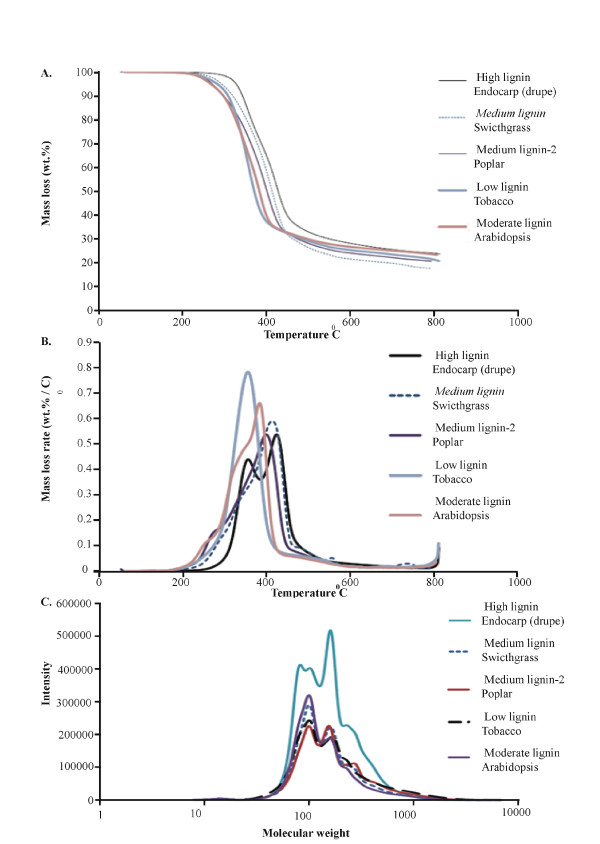
**Thermogravimetric analysis**. Thermogravimetric analysis (TGA) comparing high-lignin drupe endocarp tissue (from *Prunus persica*) with switchgrass (*Panicum virgatum*), Poplar (*Populus deltoides*), tobacco (*Nicotiana benthamiana*) and *Brassica *(*Arabidopsis thaliana*). **(A) **TGA analysis. **(B) **Differential thermogravimetric (DTG) analysis. **(C) **Gel permeation chromatography (GPC).

To establish the thermochemical differences resulting from compositional changes in the feedstocks, we used a representative endocarp feedstock (peach endocarp), a woody feedstock (*Populus spp*.), a grass feedstock (switchgrass), a moderate-lignin feedstock (*Arabidopsis*) and one low-lignin feedstock (*N. benthamiana *stem) for pyrolysis in both a thermogravimetry analyzer (TGA) and a micropyrolysis reactor (pyroprobe). We condensed out the liquid products from the micropyrolysis reactor and performed gel permeation chromatography (GPC) to analyze the liquid pyrolytic products. On the basis of the TG curves, it is apparent that peach endocarp pyrolyzed at a higher temperature in comparison with the other feedstocks (Figure 2A). Furthermore, it is evident that endocarp biomass displayed approximately 10% less weight loss at 450°C relative to other feedstocks (Figure 2A). None of the samples were volatilized above 85%, which might be due to the repolymerization of lignin residues' forming "hard coke" [4].

Differential thermogravimetry (DTG) analysis showed that endocarp biomass underwent decomposition at a higher temperature (about 450°C) compared to all other feedstocks (Figure 2B). It has been reported that hemicellulose and cellulose show DTG peaks at 268°C and 355°C, respectively [39]. Low- and moderate-lignin-containing feedstocks showed peaks at 350°C to 400°C, which might correspond to hemicellulose and cellulose peaks, whereas all medium- and high-lignin feedstocks showed peaks at higher temperatures. In addition, peach endocarp decomposition took place at two different temperatures (350°C to 400°C and 400°C to 450°C) containing two peaks, whereas the other feedstocks did not show obvious double-peaks. The decomposition temperature of the largest peak in the DTG analysis increased with the increase in lignin content (low-lignin tobacco < moderate-lignin *Arabidopsis *< medium-lignin switchgrass ≈ medium-lignin poplar < high-lignin endocarp). This result suggests that lignin content increases the pyrolysis temperature of the biomass sample. Endocarp biomass also showed a lower rate of mass loss (in wt%/°C) compared to other samples (Figure 2B). The rate of loss was indirectly proportional to the lignin content. Peach endocarp, with the highest lignin content, showed the lowest mass loss rate, and tobacco (*N. benthamiana*), with the lowest lignin content, showed the highest loss rate in this study. Also, low-lignin samples showed early thermal decomposition compared to moderate, woody and endocarp biomass samples (Figure 2B).

GPC analysis was performed to characterize the molecular weight distributions of different liquid pyrolytic products (Figure 2C). The molecular weight distributions from the GPC results are mainly from the phenolic compounds derived from lignin, because these compounds have high sensitivity in the UV detector. GPC analysis of peach endocarp biomass showed a very distinct high-intensity peak at a molecular weight of 160 compared to the other feedstocks studied (Figure 2C). Also, peach endocarp biomass showed continuous higher intensity compared to other feedstocks across all molecular weights. The intensity of peaks at molecular weights of 100 and 160 are different in endocarp biomass compared to other feedstocks. For peach endocarp biomass, the highest intensity peak corresponded to the molecular weight of 160, with the peak molecular weight of 100 being of lower intensity, whereas the opposite was true for the other feedstocks. Although we are unable to unambiguously assign these peaks to specific compounds, the observed differences at least indicate that compositionally there are significant differences between peach endocarp lignin and lignin in the other biomass types.

### Analysis of lignin composition using pyrolysis gas chromatography-mass spectrometry

**Figure 3:**
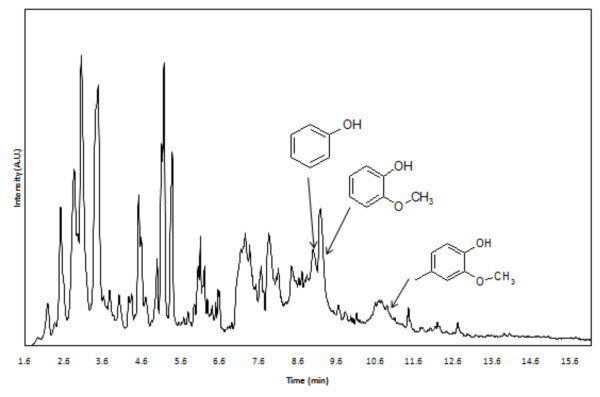
**Pyrogram results of pyrolysis of switchgrass at 650°C**.

On the basis of the results of the GPC curve, we hypothesized that drupe endocarp feedstocks might produce products different from other types of whole biomass in fast pyrolysis because of the changes in the organization of the plant cell wall. To test this postulate, we employed pyrolysis gas chromatography-mass spectrometry (Py-GC-MS), which utilizes a microscale quartz reactor inserted into a platinum wire probe capable of heating to high temperatures at extremely fast rates. This pyroprobe is directly coupled to a GC-MS instrument through a transfer line, allowing rapid analysis. Our results document that Py-GC-MS is an effective means by which to identify the differences in biomass composition and structure in selected drupe endocarp tissue (walnut, coconut and olive) rather than in a dedicated bioenergy crop (switchgrass) (see Table 3 for selected marker compounds for both lignin and holocellulosic fractions with their retention times and sources as they appear in the pyrograms). Lignin extracted from each of these sources and analyzed by Py-GC-MS revealed a variety of pyrolytic products, including methoxyphenols and other aromatic compounds derived from the monomeric units coumaryl, coniferyl and sinapyl alcohols within the lignin structure (Table 3). These compounds displayed retention times in excess of 8.8 minutes. Holocellulosic pyrolytic products include hydroxyacetaldehyde, furan derivatives, furfural, acetic acid and other short-chain oxygenated compounds. These compounds appear in pyrograms at retention times up to 8.8 minutes (Table 3 and Figure 3).

**Table 3:** Select compounds identified in pyrograms obtained from biomass and lignin pyrolysis

Compound	Retention time (minutes)	Source
Benzene	2.6	Lignin
Hydroxyacetaldehyde	2.8	Lignin + holocellulose
Acetic acid	3.0	Lignin + holocellulose
Toluene	3.4 to 3.5	Lignin
1-hydroxy-2-propanone	3.5	Lignin + holocellulose
Acetone	5.2	Lignin + holocellulose
Furfural	5.4 to 5.9	Lignin + holocellulose
2(5H)-furanone	7.9	Lignin + holocellulose
Phenol	8.9 to 9.1	Lignin
2-methoxyphenol	9.1 to 9.2	Lignin
2-methylphenol	9.4 to 9.5	Lignin
2,6-dimethylphenol	9.7 to 9.8	Lignin
4-methylphenol	10.1 to 10.5	Lignin
2-methoxy-4-methylphenol	10.7 to 11.2	Lignin
4-ethyl-2-methoxyphenol	11.8 to 12.1	Lignin
2-methoxy-4-vinylphenol	12.4 to 13.1	Lignin
2,6-dimethoxyphenol	13.2 to 13.6	Lignin
2-methoxy-4-(1-propenyl) phenol	14.2	Lignin
2-methoxy-4-(2-propenyl) phenol	12.6	Lignin
Vanillin	14.4	Lignin

Consistent with the insoluble lignin content of switchgrass being lower than that documented in endocarp biomass, switchgrass displayed the least amount of lignin pyrolytic products and a higher amount of holocellulose-based pyrolytic products that did drupe endocarp tissue (Table 4). Specifically, higher amounts of acetic acid, 1-hydroxy-2-propanone, acetone and furfural were obtained from switchgrass than from endocarp tissue (Table 4 and Figure 3). By contrast, compared to products from drupe endocarp tissue, lignin-based pyrolytic products such as phenol, 2-methoxyphenol, 2-methylphenol, 2-methoxy-4-methylphenol and 4-ethyl-2-methoxyphenol are generated at higher yields. Particularly among drupe endocarp samples, coconut showed five times more phenol content than walnut and olive endocarp did, indicating heterogeneity in lignin composition.

**Table 4:** Comparison of compounds (% area on pyrogram) produced from whole biomass pyrolysis at 650°C

	Feedstock				
	
Compound	Switchgrass	Walnut (DE)	Olive (DE)	Coconut (DE)	Peach (DE)
Hydroxyacetaldehyde	5.11	3.36	3.91	3	3.89
Acetic acid	18.41	11.77	12.96	12.49	10.78
1-hyroxy-2-propanone	16.18	5.36	6.76	4.52	3.97
Acetone	10.82	5.01	5.2	4.37	3.49
Furfural	8.42	3.31	4.08	2.86	3.86
2(5H)-furanone	3.93	3.32	2.6	2.27	0.89
Phenol	0	3.84	3.76	15.96	3.28
2-methoxyphenol	3.31	10.66	11.65	7.4	3.65
2-methylphenol	0	1.41	1.73	1.44	0.48
4-methylphenol	0.89	1.9	0	1.61	2.18
2-methoxy-4-methylphenol	0	8.83	4.9	3.9	3.98
4-ethyl-2-methoxyphenol	0	2	0.6	0.9	1.04
2-methoxy-4-vinylphenol	1.02	1.37	0	1.56	7.54
2,6-dimethoxyphenol	0	0	0	0	0
2-methoxy-4-(2-propenyl)-phenol	0	0	0	0	3.88
Vanillin	0	0	0	0	0
Sum lignin	5.21	30.01	22.63	32.75	26.03

DE = drupe endocarp.

**Figure 4:**
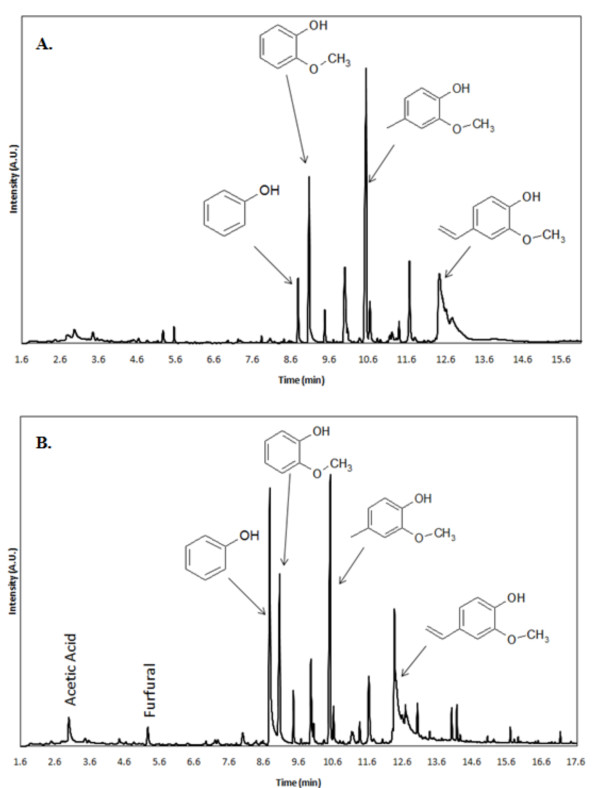
**Comparison of pyrograms results of pyrolysis of purified lignin samples**. Comparison of pyrogram results of pyrolysis of purified lignin samples at 650°C. **(A) **Pyrogram results from walnut shell. **(B) **Pyrogram results from coconut shell.

To further investigate the lignin deconstruction products, extracted lignin from the individual samples was analyzed by Py-GC-MS. The results show proportional differences among purified lignin from feedstocks, particularly between switchgrass and endocarp biomass (Table 5). Compared to endocarp lignin, switchgrass lignin showed higher amounts of acetic acid, toluene, furfural and 4-methylphenol, as well as lower or undetectable amounts of 4-ethyl-2-methoxyphenol, 2-methoxy-4-vinylphenol and 2-methoxy-4-(2-propenyl)-phenol. Walnut and olive endocarp showed comparable quantities of different lignin pyrolytic compounds. Coconut endocarp lignin showed major differences from walnut and olive endocarp lignin in producing a strikingly higher amount of phenol and a lower amount of 2-methoxy-4-methylphenol (see Figures 4A and 4B). Coconut shell lignin contained unique signature compounds among the analyzed feedstocks, such as 2,6-dimethoxyphenol, 2-methoxy-4-(2-propenyl)-phenol and vanillin, but less 2-methoxy-4-(1-propenyl)-phenol compared to walnut shell and olive stone lignin.

**Table 5:** Comparison of compounds (% area on pyrogram) produced from purified lignin pyrolysis at 650°C

	Feedstock				
Compound	Switchgrass	Walnut DE	Olive DE	Coconut DE	Peach DE
Benzene	0.51	0	0	0	0.47
Acetic acid	6.32	2.47	4.26	2.97	3.26
Toluene	2.74	0.79	0.79	0.43	1.65
Propanoic acid	0.65	0	0	0	0
Xylene	0.45	0.37	0.34	0.16	0.45
Furfural	2.46	0.97	1.11	1	1.36
Phenol	8.23	3.67	1.06	18.41	2.71
2-methoxyphenol	9.93	11.41	11.03	9.64	11.63
2-methylphenol	2.35	1.89	1.4	2.13	2.02
2,6-dimethylphenol	0.15	0.12	0.17	0.21	0.18
4-methylphenol	11.53	8.25	3.7	4.09	6.46
2-methoxy-4-methylphenol	20.51	21.63	21.02	12.82	26.4
4-ethyl-2-methoxyphenol	0	5.5	5.7	3.86	5.16
2-methoxy-4-vinylphenol	6.28	18.39	12.39	19.22	6.93
2,6-dimethoxyphenol	0	0	0	1.56	0
2-methoxy-4-(1-propenyl)-phenol	0	0	0	2.14	0
2-methoxy-4-(2-propenyl)-phenol	0	10.88	14.45	6.46	13.2
Vanillin	0	0	0	0.17	0

DE = drupe endocarp.

It should also be noted that different amounts and types of lignin pyrolytic products are seen when the lignin is pyrolyzed separately from the hollocellulose fraction for each biomass sample. This indicates that the pyrolysis process is altered depending on the variation in linkages between the individual biopolymers. Moreover, pyrolysis of coconut shell lignin showed that pyrolysis of the lignin fraction also creates a small amount of acetic acid and furfural (Figure 4b). We believe that this result arose from residual hemicellulose bound to the lignin fraction (see Methods). In summary, pyrograms of purified lignin derived from coconut endocarp documented proportionally greater amounts of phenol production than those of olive or walnut endocarp, whereas the amount of 2-methoxy-4-methylphenol was less abundant. Coconut shell lignin pyrolysis also produced proportionately more 2,6-dimethoxyphenol than all other biomass samples (Table 4). Given that 2,6-dimethoxyphenol is considered to be a marker for sinapyl alcohol [9], this result suggests that coconut shells may contain higher amounts of the sinapyl alcohol monomer in the lignin structure.

## Discussion

Lignification of plant tissues occurs with a greater degree of spatial regulation than cellulose deposition. For instance, only certain cell types, such as xylem vessels, are lignified, whereas all plant cells synthesize cellulose [11,40]. When selecting feedstocks to examine, we attempted to cover a broad range of tissue types in order to encompass this variation in cell-wall composition. On the basis of compositional profiling, we illustrate herein that drupe endocarp agricultural by-products from the fruit and nut production industries meet the criterion of high feedstock homogeneity with respect to lignin content, with up to 50% of the cell wall being composed of lignin. Consistent with lignin's displaying a higher heating value compared to cellulose [41], we have documented that drupe endocarp biomass as a bioenergy feedstock displayed the highest net energy density of any biomass feedstock examined. Higher energy density, coupled with undetectable or very low ash content compared to other feedstocks, suggests that drupe endocarp biomass is an attractive feedstock the use of which can reduce costs associated with cleaning boilers and gasifiers [42] (Tables 1 and 2).

In this study, we examined the composition of representative endocarp biomass and a dedicated bioenergy crop (switchgrass) by Py-GC-MS and found that variation in lignin composition results in substantial differences in product yield and species. Our Py-GC-MS data (Tables 3 and 4) are consistent with our GPC, TGA and DTG analyses (Figure 2), which illustrate modest differences in the deconstruction characteristics of high-lignin endocarp relative to low-lignin biomass. The most striking difference observed during biomass deconstruction is illustrated by the DTG results, which show that a high-intensity peak at a molecular weight of 160 arose in the deconstruction profile for endocarp biomass. Overall the Py-GC-MS results show variation in the amounts of pyrolytic products, thereby imparting distinct compositional properties to the bio-oil from different sources. The highest yield change in endocarp-derived chemicals relative to switchgrass arose in the form of phenol, 2-methoxyphenol, 2-methylphenol, 2-methoxy-4-methylphenol and 4-ethyl-2-methoxyphenol. Admittedly, modulating the yield of specific deconstruction products will have economic benefit only when the market demand for these chemicals is sufficiently valuable. Although in this study we aimed to examine the impact of variable lignin content and cell-wall composition on overall pyrolytic deconstruction products, we note the active, ongoing research seeking to improve the pyrolytic process by expanding the range of catalysts for process-targeted, catalytic, fast pyrolysis [43,44]. Ultimately, tailoring biomass composition as well as the deconstruction chemical engineering process offers potential in improving fuel and chemical synthesis. In this context, approximately 13% of crude fossil oil is used for the production of chemicals [45], suggesting translational applications of biomass-sourced bioproducts. Several industries are currently producing bio-oil and other biobased materials by using fast pyrolysis (for example, Avello Bioenergy (Boone, IA, USA), DynaMotive Energy Systems Corp (Vancouver, BC, Canada), Renewable Oil International LLC (Florence, AL, USA), etc). However, owing to the thermal instability, high oxygen content, high viscosity and high acidity of bio-oils, hurdles still need to be cleared before bio-oils can be used as a direct replacement for gasoline without upgrading [44,46]. In addition to fuel replacement, substitutes for oxygenated bulk chemicals, such as ethylene glycol, propylene glycol and acetone, must arise from biomass deconstruction if fossil fuels are to be replaced [47-51]. To develop drop-in fuels and chemicals, a major research thrust which includes the conversion of lignin is needed.

## Conclusions

Compositional analysis and the bioenergy conversion potential of these high-lignin feedstocks represent an underexploited source of bioelectricity and hydrocarbon-based renewable energy or biobased chemicals. Furthermore, the data reported herein document that different cell-wall composition results in different pyrolytic breakdown products and yields. High-lignin drupe endocarp feedstocks appear to be a source of the renewable production of phenol, 2-methoxyphenol, 2-methylphenol, 2-methoxy-4-methylphenol and 4-ethyl-2-methoxyphenol. Despite the examples of deconstruction products documented herein, an overarching problem with utilizing lignin associated with its structural diversity and heterogeneity will make it challenging to produce catalysts for targeted pyrolytic deconstruction. A desirable biotechnological breakthrough would be to modify or simplify lignin structure in plants consisting of only one rather than three phenol alcohols or to simplify the complex, recalcitrant interunit linkages separating the lignin monomers.

## Methods

### Chemicals

All chemicals and reagents used were of analytical grade or higher. Authentic samples of organic compounds were obtained as applicable from Sigma-Aldrich (St Louis, MO, USA), FMC BioPolymer (Philadelphia, PA, USA), Fisher Scientific (Pittsburgh, PA, USA), Riedel-de Haën (Seelze, Germany) and BDH Merck Ltd (Poole, UK)

### Feedstocks

Feedstocks were selected from *Arabidopsis *(*Arabidopsis thaliana*) (model plant stem), switchgrass (*P. virgatum*) (biofuel monocot plant, aerial), poplar (*P. trichocarpa *and *P. deltoides*) (biofuel dicot plants, stems), tobacco (*N. benthamiana*) (stem), peach drupe endocarp (*P. persica*), coconut drupe endocarp (*C. nucifera*), olive drupe endocarp (*O. europaea*) and walnut drupe endocarp (*Juglans spp*.) (agricultural by-products: drupe fruit). Poplar biomass was collected from The University of Kentucky Energy Crop repository and comprised coppiced two-year-old plants that were approximately 6 m in height. *Arabidopsis thaliana *and *N. benthamiana *were grown in greenhouse conditions under long-day 16:8-hour light:dark photoperiods and harvested at maturity. Peach and black walnut endocarp biomass was collected from *P. persica *grown at The University of Kentucky Horticulture Research Farm, Lexington, KY. Fresh coconut and olive endocarp biomass sourced at a commercial vegetable outlet was obtained and manually cleaned of fruit flesh. Feedstocks were dried at 37°C for seven days and ground to a 1-mm homogeneous size using an Arthur H Thomas Co Scientific grinder (Philadelphia, PA, USA) prior to analysis.

### Quantitative assessment of endogenous lignin, cellulose and calorific content for given feedstocks

Acid-soluble and acid-insoluble lignin contents of different feedstocks were measured according to the method published by the National Renewable Energy Laboratory (NREL, Golden, CO, USA) protocol (http://www.nrel.gov/biomass/pdfs/42618.pdf). Briefly, 300 mg of the biomass contained in a glass tube were hydrolyzed using 3 ml of 72% sulfuric acid in a water bath at 30°C for one hour with intermittent stirring every 15 minutes. The tubes were removed from the water bath, and the acid was diluted to a 4% concentration by adding 84 ml of deionized water, after which the contents were autoclaved for one hour at 121°C. Acid-soluble lignin content was calculated by measuring sample optical density at 240 nm using a spectrophotometer (Thermo Scientific Biomate 3; Thermo Fisher Scientific, Waltham, MA, USA) as described in the NREL protocol. For acid-insoluble lignin content determination, the autoclaved samples were filtered through crucibles. Sample weight was taken after drying the sample at 105°C overnight. The crucibles were placed into a furnace, and the temperature was gradually allowed to reach 575°C. After four-hour incubation at 575°C, the furnace temperature was set to 105°C, and the furnace was allowed to slowly reach the set temperature. The samples were moved to a desiccator until they reached room temperature, and the weight loss was recorded and used for the calculation of acid-insoluble lignin content. The cellulose content of each feedstock was measured spectrophotometrically (Thermo Fisher Biomate 3) on homogeneous samples using a method described previously [52]. Calorific values were determined by using a bomb calorimeter.

### Thermogravimetry, differential thermogravimetry and gel permeation chromatography of high-lignin feedstocks

Pyrolysis in a TGA was performed at a temperature range of 50°C/minute to 800°C/minute as described previously [4]. The samples were preheated to 110°C during the initial 30 minutes to remove the moisture content. Next the samples were heated to a final temperature at a rate of 150°C/minute. Ultra-high-purity helium was used as the sweep gas with a 100 ml/minute flow rate at atmospheric pressure. GPC was performed using a Shimadzu HPLC system with a UV detector (frequency 254 nm) (Shimadzu Corp, Kyoto, Japan). A MesoPore column (1113-6325; Varian, Inc, Cary, NC, USA) was used with stabilized tetrahydrofuran (THF) as the mobile phase flowing at 0.5 cm^3^/minute. Samples for GPC were prepared by condensing the pyrolysis vapor in the pyroprobe, coupled with a condenser trap designed in-house, using the following reaction conditions: temperature 600°C, heating rate 1,000°C/second and reaction time 240 seconds. Condensed vapors were then dissolved in THF at 1 wt% concentration. The solution was filtered through a 0.45-μm filter and used for GPC. The GPC column was standardized using polystyrene molecular weight standards in the range of 162 to 38,640 Da.

### Compositional analysis of feedstocks using Py-GC-MS

Experiments were performed using a Pyroprobe Model 5200 (CDS Analytical, Inc, Oxford, PA, USA) connected to an Agilent 7890A GC gas chromatograph system with an Agilent 5975C Series GC/MSD detector (Agilent Technologies, Inc, Santa Clara, CA, USA). The pyroprobe was run in trap mode without the use of a reactant gas, and we utilized a sorbent tube containing Tenax TA adsorbent resin (Alltech Associates Inc., Deerfield IL). Pyrolysis was conducted at 650°C (1,000°C/second heating rate) for 20 seconds under helium gas. The valve oven and transfer lines were maintained at 325°C. The column used in the gas chromatograph was a DB1701 (30 m × 0.25 mm × 0.1 μm; Agilent Technologies, Inc), and the temperature program was as follows: 50°C for one minute, ramp to 280°C at 10°C/minute and hold for five minutes. The flow rate was set to 1 ml/minute using helium as the carrier gas. The inlet and auxiliary lines were maintained at 325°C and 310°C, respectively, and the MS source was set at 69 eV. The GC-MS was calibrated for a number of phenolic compounds, including phenol, 2-methoxyphenol, 2-methoxy-4-methylphenol and 2-methoxy-4-vinylphenol.

Prior to analysis, a 1-cm-long quartz cell packed with only quartz wool was heated to 1,000°C using the pyroprobe and then run in a blank pyrolysis experiment to ensure that the cell and the pyroprobe system were clean. Approximately 1 mg of finely ground biomass was then placed inside the quartz cells packed with quartz wool. The quartz wool was then packed on top of the sample inside the cell, and the cell was placed into the pyroprobe. The pyroprobe was heated to 100°C for 10 seconds *ex situ *twice to dry the samples. The samples were then pyrolyzed according to the procedure previously described. Biomass samples analyzed included degummed switchgrass, walnut shells, coconut shells, olive stones and the lignin extracted from each type of biomass.

### Lignin sample preparation for Py-GC-MS

A modified lignin extraction method was employed to extract lignin from different biomass samples for further Py-GC-MS. The NREL protocol for the determination of lignin and carbohydrates requires the use of 72% sulfuric acid. Although it is effective for quantifying the ultimate amounts of lignin present in the samples, strong acid treatment fundamentally and irreversibly alters the composition of the extracted lignin. These alterations result in degradation and recondensation of the lignin oligomers in a manner that yields a different lignin than that initially present in the biomass. We used a method modified from one described previously [53] to extract lignin by utilizing 85% formic acid with the goal of developing an easily repeatable lignin extraction method for use at room temperature that would result in minimal alterations to the initial lignin structure. Prior to pyrolysis, each biomass sample was ball-milled for 10 minutes and subsequently degummed by overnight soxhlet extraction using acetone. Lignin was extracted from the biomass by a modified formic acid treatment similar to that described by Zhang *et al*. [53]. The biomass was placed in a shaker flask with 85% formic acid at a ratio of 20 ml liquid to 1 g solid, to which 0.1 wt% of hydrogen chloride was added as a catalyst. The mixture was then heated at 60°C under constant agitation. After 24 hours, the mixture was filtered and the filtrate was reduced to dryness using a rotary evaporator. To separate the lignin and hemicellulose components, the resulting film was washed with distilled water and then filtered to recover the lignin, which was dried in an oven at 80°C.

## Abbreviations

DE: drupe endocarp; DTG: differential thermogravimetry; DW: dry weight; FW: fresh weight; GJ: gigajoule; GPC: gel permeation chromatography; MJ: megajoule; Py-GC-MS: pyrolysis gas chromatography-mass spectrometry; TG, thermogravimetry; TGA: thermogravimetric analyzer.

## Competing interests

The authors declare that they have no competing interests.

## Authors' contributions

VM carried out the sample isolation, biomass composition and energy content and drafted the manuscript. LHW, MC, SM and AP carried out the extraction of lignin, developed the pyroprobe assays and drafted the manuscript. JJ and GH generated the GPC, TGA and DTG curves for thermochemical deconstruction. SD conceived the study, performed the statistical analysis and drafted the manuscript. All authors read and approved the final manuscript.
